# Acute systemic inflammation reduces both carotid and aortic wave reflection in healthy adults

**DOI:** 10.14814/phy2.14203

**Published:** 2019-08-11

**Authors:** Elizabeth C. Schroeder, Wesley K. Lefferts, Thessa I. M. Hilgenkamp, Bo Fernhall

**Affiliations:** ^1^ Integrative Physiology Laboratory, College of Applied Health Sciences University of Illinois at Chicago Chicago Illinois

**Keywords:** Acute inflammation, hemodynamics, wave reflection

## Abstract

Acute inflammation increases the risk of cardiac and cerebrovascular events, possibly related to alterations in the hemodynamic load. Wave reflection at the aorta and carotid provides insight into downstream vascular changes and hemodynamic load at the heart and brain. Acute inflammation has been suggested to reduce wave reflection via downstream vasodilation; however, this is not firmly established and has only been investigated at the aorta. We sought to explore the effect of acute inflammation on aortic and carotid hemodynamics in healthy, young adults. Pressure waveforms were collected via radial and carotid applanation tonometry in 23 adults (26 ± 4 years) before and 24 h after a typhoid vaccination. Waveforms were calibrated to brachial mean and diastolic pressure, and waveform separation analyses (WSA) were performed, yielding augmentation index, reflection index, time to reflection (Tr), forward (Pf) and reflected (Pb) wave magnitude, and pulse wave velocity. Arterial diameters and carotid stiffness were measured via ultrasonography. Acute inflammation reduced wave reflection at 24 h in both the aorta and carotid (*P* < 0.05) without changes in mean pressure. WSA did not reveal independent changes in Pf, Pb, or Tr (*P* > 0.05). Arterial stiffness did not change; however, brachial and carotid artery diameters increased. Acute inflammation reduces wave reflection in the aorta and carotid artery in young adults, potentially due to downstream/peripheral vasodilation. Reduced aortic wave reflection did not disturb the cardiac workload; however, reductions in carotid wave reflection may render the brain vulnerable to pulsatile hemodynamics. These findings may have implications for cardiac and cerebrovascular risk during acute inflammation.

## Introduction

Acute inflammation is associated with an increased risk of cardiovascular and cerebrovascular events, especially within the first week following infection (Syrjänen et al. 1988; Smeeth et al. 2004; Corrales‐Medina et al. 2010, 2015). This increased risk and vulnerability of the heart and brain during acute inflammation may reflect changes in hemodynamic load from altered peripheral wave reflections.

Left ventricular contraction generates a forward traveling pressure/energy wave that can be partially reflected by downstream bifurcations and/or changes in vasomotor tone (Chirinos and Segers, 2010a; Townsend et al. 2015). Vasodilation reduces, and vasoconstriction increases, the portion of the forward wave that is reflected. This reflected wave travels back upstream and combines with the subsequent forward wave, altering central hemodynamics. A larger reflected wave may combine with and augment the forward wave, thereby increasing systolic pressure (Nichols et al. 2008; Avolio et al. 2009). Even in the absence of a larger reflected wave, faster traveling reflected waves (as seen with increasing arterial stiffness) may arrive earlier (i.e., late systole) and augment systolic pressure (Nichols, 2005). The impact of wave reflections on target organs, however, is dependent on their location in the central vasculature.

Increased wave reflections in the ascending aorta can augment central pressure and increase cardiac workload and perfusion balance (Chirinos and Segers, 2010b; Namasivayam et al. 2011). Increased aortic wave reflection and forward wave magnitude have both been implicated in increased mortality and morbidity (Zamani et al. 2014). In the carotid, however, increases in wave reflection from the cerebral circulation may increase the pulsatile pressure in the carotid artery but decrease the pulsatile flow transmission into the brain (Nichols et al. 2011; Tarumi et al. 2014). Moreover, emerging data suggest wave reflections from below the carotid‐aorta interface may become forward traveling waves in the carotid and increase pulsatile hemodynamics transmitted into the brain (Mynard et al. 2017; Hashimoto et al. 2018). As such, examining aorta and carotid wave reflections may provide insight into downstream vascular changes and central hemodynamic load at target organs such as the heart and brain.

Acute inflammation has been suggested to reduce wave reflection via peripheral vasodilation (Vlachopoulos et al. 2005). This reduction in wave reflection has not been firmly established (Wallace et al. 2010; Rathod et al. 2017) and has only been investigated at the level of the aorta, with little focus on carotid wave reflections or the potential interaction between wave reflections above and below the heart. Additionally, previous literature regarding acute inflammation and wave reflection has relied on augmentation index, instead of more robust techniques like wave separation analyses, which may provide limited insights in young adults with negative augmentation (Hughes et al. 2013). Wave separation analysis provides insight regarding independent forward and reflected wave magnitude and timing and is a more robust method to interrogate the effect of acute inflammation on wave reflections and hemodynamics in the central vasculature. Therefore, the purpose of this study was to explore the effect of acute inflammation on aortic and carotid hemodynamics in healthy, young adults. We hypothesized acute inflammation would reduce wave reflection in the aorta and carotid artery due to peripheral vasodilation.

## Methods

### Ethical approval

This study was approved by the Institutional Review Board at the University of Illinois at Chicago (#2017‐0560) and conformed to the guidelines set forth by the Declaration of Helsinki, except for registration in a database. All participants provided written informed consent prior to participation.

### Participants and design

Healthy, young adults aged 18–35 years were recruited from the local university for participation in this experimental study design. Exclusion criteria included antioxidant or vitamin supplementation; anti‐inflammatory medications within the previous 2 weeks; typhoid vaccination within 2 years or a prior adverse reaction; illness within 2 weeks prior to testing; smoking; pregnancy; a body mass index >35 kg/m^2^; any known cardiovascular, metabolic, or inflammatory disease; or current use of blood pressure medications or other drugs influencing cardiovascular outcomes.

Participants arrived to the laboratory having abstained from caffeine, alcohol, and physical activity for ≥24 h and fasted for ≥10 h. All females were tested during the first 7 days of their menstrual cycle or during their placebo week if taking oral contraceptives (*n* = 3). Height and weight were measured and body mass index (BMI) was calculated (kg/m^2^).

Each participant completed two study visits. The study visits were completed on consecutive days at the same time of day to avoid diurnal variation and to ensure 24 h between measures. For each visit, a fasting blood sample was collected and then participants rested quietly for 10 min in the supine position in a temperature‐controlled room before all vascular and hemodynamic measures were conducted. At the end of the first visit, the *Salmonella typhi* polysaccaharide vaccine (Typhim Vi, Sanofi Pasteur SA) was administered by a registered nurse into the nondominant arm to induce acute inflammation. Vaccinations provide a safe and controlled inflammatory response and have been previously been used during cardiovascular research (Hingorani et al. 2000; Clapp et al. 2004; Schroeder et al. 2018).

### Measures

#### Brachial blood pressure

Hemodynamic parameters (brachial systolic [SBP] and diastolic [DBP] pressure, and peripheral vascular resistance) were measured in duplicate on the right arm using an automated ambulatory blood pressure monitor (Mobil‐O‐Graph 24 PWA, I.E.M., Stolberg Germany). Mean arterial pressure (MAP) was calculated as 1/3 SBP + 2/3 DBP.

#### Aortic and carotid blood pressure and wave reflection

Pressure waveforms were collected via applanation tonometry (SphygmoCor Model EM3, AtCor Medical, Sydney, Australia) from the radial and common carotid artery in 10 s‐epochs and ensemble averaged to create representative waveforms (SphygmoCor Software Version 9). All waveforms were calibrated to brachial mean and diastolic pressure. The radial waveform was further transformed algorithmically using a validated generalized transfer function to estimate aortic pressure (Karamanoglu et al. 1993; Chen et al. 1997; Pauca et al. 2001). For quality control, all measures were completed in duplicate in which the estimated blood pressures and augmentation index needed to be within 5 mmHg and 5%, respectively.

Additional variables were calculated based on the respective aortic and carotid waveforms. Pulse pressure (PP) was calculated as SBP – DBP. Augmentation index (AIx) was used as a measure of global wave reflections, calculated as the percent of augmented pressure (difference between late and early systolic peaks of the waveform) to total PP ([P_2_−P_1_]/PP*100) and normalized to a heart rate of 75 bpm (AIx75).

Wave separation analyses were performed on the aortic and carotid waveforms to determine the forward (Pf) and reflected (Pb) wave components. This is based on the flow triangulation method of Westerhof et al. (2006) and uses a modified average‐flow waveform. Reflection index was calculated as a measure of reflection magnitude not dependent on heart rate by dividing Pb by Pf (Butlin and Qasem, 2016). Time to reflection (Tr), diastolic pressure–time integral (DPTI), and subendocardial viability ratio (SEVR) were also determined from the aortic pressure waveform as the systolic travel time of the pressure wave, an estimate of oxygen supply to the myocardium, and a measure of oxygen supply and demand, respectively.

#### Arterial stiffness

Aortic pulse wave velocity (PWV) was estimated from the aortic pressure waveform described above using the time lag between forward and reflected wave components (Qasem and Avolio, 2008). Carotid‐femoral distance for PWV was estimated by multiplying height by 0.29 (Filipovský et al. 2010).

β‐Stiffness index was used to determine the arterial stiffness of the carotid artery. The right common carotid artery was imaged longitudinally via ultrasonography approximately 1–2 cm proximal to the bifurcation (Hitachi‐Aloka Alpha 7, Tokyo, Japan). Pressure waveforms and vessel diameters were determined from 6 to 10 consecutive waves using automated wall detection echo‐tracking software. β‐Stiffness index was subsequently calculated as [ln(P_systolic_/P_diastolic_)]/[(D_systolic_−D_diastolic_)/D_diastolic_], where P_systolic_ and P_diastolic_ are carotid SBP and DBP, respectively, and D_systolic_ and D_diastolic_ are arterial diameters.

#### Arterial vasomotor tone

Using the images of the right common carotid artery collected for β‐stiffness index (described above), mean carotid diameter was calculated as 1/3 systolic diameter + 2/3 diastolic diameter.

Right brachial artery diameter was imaged via ultrasound using a high‐frequency linear array probe approximately 5 cm proximal to the antecubital fossa. Data were recorded for offline analysis with automatic edge detection software (FMD Studio Cardiovascular Suite, QUIPU, Pisa, Italy). A 30‐sec–steady‐state diameter was averaged and reported.

Resistance artery function was assessed using strain gauge plethysmography. A strain gauge was placed around the widest aspect of the forearm, and resting forearm blood flow (FBF, mL/min/100 mL tissue) was assessed by averaging six stable measures (7 sec occlusion, 8 sec deflation). Forearm vascular conductance (FVC, mL/min/100 mL tissue/100 mmHg) was calculated by dividing FBF by MAP and multiplying by 100.

### Statistical analyses

All data are reported as mean and standard deviation. Normality was assessed with the Kolmogorov–Smirnov test. Data were log transformed when necessary and reported as raw means for interpretation. All outcome variables were assessed with a repeated measures analysis of variance to determine the effect of acute inflammation. Data analyses were performed using SPSS version 24 (IBM Corporation, Armonk, New York, USA) and all *P* values are two‐sided, with an a priori α‐level of 0.05 determined to be significant.

Vlachopoulos et al. (2005) studied the effects of acute inflammation on wave reflection with a sample size of 24; however, only scarce raw data were provided for their reduction in AIx at 8 and 32 h to perform a power calculation. Since they observed a significant reduction in AIx and AIx@75, we anticipated a sample size of 23 would have sufficient power to detect changes in global wave reflection.

## Results

Eighty‐one individuals were screened for eligibility. Of those, 29 individuals were excluded based on study criteria, 24 chose not to participate due to the time commitment or scheduling, and five individuals were unwilling to receive the vaccine. Twenty‐three young, healthy participants (12 male, 11 female; 9 White, 2 African‐American, 3 Asian, 3 Latino, 6 Indian) completed the acute inflammation protocol. No participants had any adverse events in response to the vaccine; however, a small number of participants noted some muscle soreness at the site of the injection. Participants’ age and body mass index were 26 ± 4 years and 22.7 ± 3.2 kg/m^2^, respectively. Acute inflammation was evident at 24 h following the vaccine with increases in CRP and IL‐6 (*P* < 0.01; 1.11 ± 2.0 to 2.81 ± 3.79 mg/L and 1.12 ± 0.53 to 2.38 ± 1.33 pg/mL, respectively).

**Figure 1:**
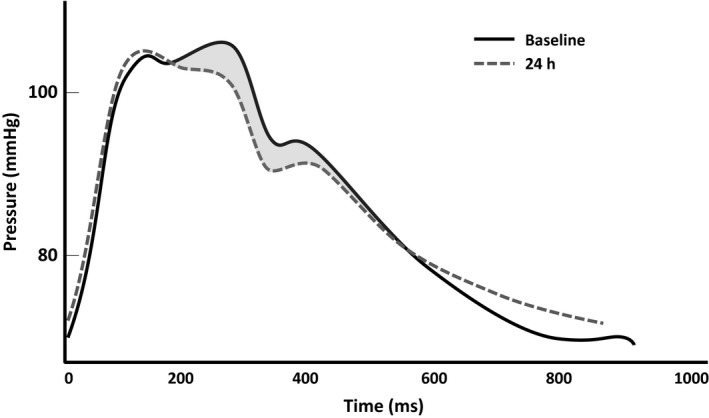
Representative carotid pressure waveforms obtained from a single participant before and during acute inflammation.

**Figure 2:**
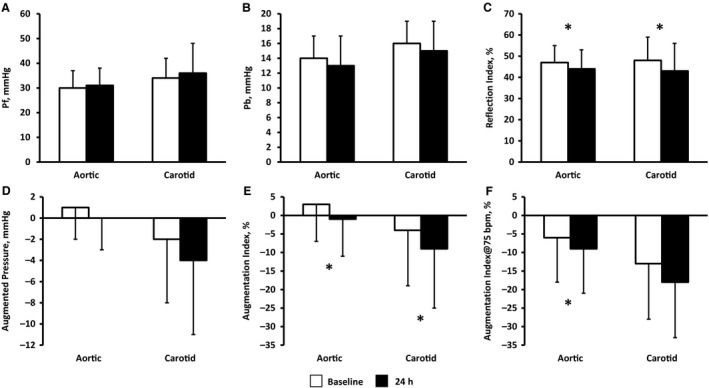
Changes in wave reflection indices at baseline and during acute inflammation. *time effect, *P* < 0.05.

Brachial mean (86 ± 9 to 85 ± 7 mmHg) and diastolic (71 ± 8 to 70 ± 7 mmHg) pressure, and heart rate (56 ± 11 to 57 ± 10 bpm) were unchanged at 24 h (>0.05). Aortic and carotid blood pressure were also maintained at 24 h (Table 1, *P* > 0.05). However, acute inflammation altered wave reflections (Figure 1) with reductions in aortic and carotid AIx (Figure 2, *P* < 0.05). After adjusting for heart rate, the reduction in aortic AIx remained (AIx@75 *P* = 0.048); however, the significant reduction in carotid AIx was eliminated (AIx@75 *P* = 0.09). Aortic DPTI was reduced during acute inflammation (Table 2, *P* = 0.04); however, SEVR was maintained (*P* = 0.23). Despite the alterations in augmentation index, wave separation analyses did not reveal independent increases in forward wave or decreases in reflected wave magnitude (*P* > 0.05). Reflection index, however, significantly decreased in both the aorta and carotid during acute inflammation (*P* < 0.05).

**Table 1:** Effect of acute inflammation on aortic and carotid hemodynamics

	Aortic			Carotid		
	Baseline	24 hours	*P*‐value	Baseline	2 hours	*P*‐value
Systolic blood pressure, mmHg	104 ± 10	104 ± 8	0.88	107 ± 11	107 ± 10	0.98
Diastolic blood pressure, mmHg	72 ± 8	71 ± 7	0.23	71 ± 8	70 ± 7	0.26
Mean arterial pressure, mmHg	86 ± 9	85 ± 7	0.31	86 ± 9	85 ± 7	0.29
Pulse pressure, mmHg	32 ± 6	33 ± 8	0.21	36 ± 7	37 ± 8	0.39

**Table 2:** Effect of acute inflammation on arterial stiffness and vasomotor tone.

	Baseline	24 hours	*P*‐value
Heart rate, bpm	56 ± 11	57 ± 10	0.20
Brachial diameter, mm	3.79 ± 0.76	3.86 ± 0.76	0.055
Carotid diameter, mm	6.44 ± 0.72	6.55 ± 0.69	0.04
Pulse wave velocity, m/s	6.7 ± 0.6	6.8 ± 0.6	0.41
Carotid β‐stiffness, au	4.2 ± 1.2	4.3 ± 1.2	0.65
Resting FBF, mL/min/100 mL tissue	2.3 ± 0.7	2.5 ± 0.7	0.34
Resting FVC, mL/min/100 mL tissue/100 mmHg	2.76 ± 0.89	2.95 ± 0.90	0.37
Peripheral vascular resistance, s*mmHg/mL	1.16 ± 0.26	1.09 ± 0.23	0.22
Aortic DPTI, au	3375 ± 469	3285 ± 409	0.04
SEVR, au	196 ± 49	187 ± 50	0.23

DPTI, diastolic pressure–time integral; FBF, forearm blood flow; FVC, forearm vascular conductance; SEVR, subendocardial viability ratio.

Acute inflammation did not alter estimated pulse wave velocity (Table 2, *P* = 0.41) or carotid β‐stiffness index (*P* = 0.65). Time to reflection was also unaltered in both the aorta (158 ± 16 to 161 ± 20 ms) and carotid artery (180 ± 25 to 185 ± 26 ms). Brachial and carotid artery diameter tended to increase from 3.79 ± 0.76 mm at baseline to 3.86 ± 0.76 mm at 24 h (*P* = 0.055) and 6.44 ± 0.72 mm at baseline to 6.55 ± 0.69 mm at 24 h (*P* = 0.04), respectively. Finally, acute inflammation‐induced modest reductions in peripheral vascular resistance (*P* = 0.22) and small increases in resting FBF and FVC, although not statistically significant (*P* > 0.05).

## Discussion

This study sought to determine the effect of acute inflammation on aortic and carotid hemodynamics in healthy, young adults. Our results suggest that acute inflammation reduces wave reflection (assessed by AIx and RIx) in both the aorta and carotid artery despite no alterations in central blood pressure. These reductions in wave reflection may be related to inflammation‐induced increases in arterial diameters. While the indices of cardiac work/perfusion balance were preserved during acute inflammation, the reduction in carotid wave reflection may render the cerebrovasculature vulnerable to pulsatile flow. These findings may therefore have implications for cardiac and cerebrovascular risk during acute inflammation.

### Aortic and carotid hemodynamics

We observed significant reductions in aortic AIx, confirming previous work during acute inflammation in young adults (Vlachopoulos et al. 2005). However, to our knowledge, we are the first to report reductions in carotid AIx. AIx is a global marker of wave reflection which does not supply information on alterations in either the magnitude or timing of the reflected waves. Expanding on the work by Vlachopoulos et al. (2005), we performed wave separation analyses to investigate the magnitude and timing of forward and reflected waves at both the aorta and carotid artery.

Acute inflammation significantly decreased both aortic and carotid reflection index (assessed as the ratio of forward to reflected wave magnitude). This effect stemmed from modest, nonstatistically significant increases in forward and decreases in reflected wave magnitudes at the carotid and aorta during acute inflammation. This net change in wave reflection may reflect subtle alterations in cardiac contractility and downstream vasomotor tone. Indeed, the latter of these potential contributors aligns with the increases we observed in brachial and carotid artery diameters. As wave reflection and resistance is largely governed by small resistance arteries, we also observed slight but nonsignificant reductions in peripheral vascular resistance and small, nonsignificant increases in FBF and FVC. Our data support the notion first put forth by Vlachoupoulos et al. (2005) that changes in peripheral/downstream vasomotor tone may contribute to reductions in wave reflection during acute inflammation. Thus, reductions in wave reflection during acute inflammation likely stem from an accumulation of several physiological processes rather than one single factor.

Arterial stiffness impacts the speed and timing of wave reflections as a stiffer vessel allows the pressure wave to travel faster, and arrive earlier in the cardiac cycle (Laurent et al. 2006). We noted no change in aortic and carotid stiffness, or time to reflection during acute inflammation in our study. This suggests the observed reductions in global wave reflection (AIx) in our study may stem primarily from changes in wave magnitude, rather than arterial stiffness and timing. Vlachoupoulos et al. (2005) previously reported increases in aortic PWV in combination with a reduction in AIx during inflammation; however, we were unable to replicate this finding. One potential explanation for the differences in the aortic PWV response to acute inflammation may be the method of measurement. Although our PWV estimated using wave separation analyses is a validated measure (Qasem and Avolio, 2008), Vlachoupoulos et al (2005) used the gold standard carotid‐femoral PWV.

### Implications for end organ hemodynamics

At the level of the heart, wave reflections may alter cardiac work and perfusion balance. Our study population had a negative augmentation index at baseline and little to negative augmented pressure, thus inflammation‐induced reductions in wave reflection did not change central systolic blood pressure. While systolic work was unaltered, reduced wave reflection may also impact diastolic perfusion. Myocardial perfusion occurs during diastole when the heart is relaxed, which is partially dependent on the reflected wave returning from the periphery (Nichols, 2005). Our reductions in aortic wave reflection were accompanied by a concomitant reduction in DPTI during acute inflammation. Despite reductions in DPTI, there was no imbalance between oxygen supply and demand to the myocardium, as SEVR was maintained during acute inflammation. Ultimately, the data suggest acute inflammation may alter the indices of diastolic perfusion but does not disturb work/perfusion balance in young, healthy adults.

Wave reflections at the carotid artery stem from changes in downstream (i.e., cerebral) vasomotor tone (Bleasdale et al. 2003) and may help modulate brain blood flow pulsatility. Reductions in wave reflection in the carotid artery may render the cerebrovasculature vulnerable to the detrimental effects of increased cerebral flow pulsatility. Wave reflection theory explains that reflected pressure waves augment pressure but attenuate forward flow (Nichols et al. 2011). The reduction in carotid wave reflection during acute inflammation may reduce systolic pressure augmentation (if present) and simultaneously reduce the “braking/deceleration” effect of wave reflection on blood flow being transmitted into the brain. Wave reflection is often viewed as a negative physiological phenomena; however, increased wave reflection plays a critical role in transforming pulsatile flow to steady flow at end organs by reducing the amplitude of the transmitted flow waveform (de Roos et al. 2017). Thus, moderate acute inflammation may attenuate protection against cerebral flow pulsatility afforded by carotid wave reflections. Indeed, during severe acute inflammation such as sepsis, increased cerebral pulsatility has been observed (de Azevedo et al. 2017). Our data suggest that even moderate bouts of acute inflammation may alter cerebrovascular defenses against cerebral blood flow pulsatility. Whether this translates to changes in cerebral pulsatility during moderate acute inflammation is unclear and requires additional research.

### Implications for overall risk

Acute inflammation transiently increases the risk of cardiovascular and cerebrovascular events (Smeeth et al. 2004; Corrales‐Medina et al. 2010, 2015). This risk is not only evident in an older population, but a preceding infection has been identified as an underestimated risk factor for ischemic brain infarction in individuals under 50 years of age (Syrjänen et al. 1988). Given the influence of wave reflection on cardiac workload and flow pulsatility in the sensitive cerebrovasculature, alterations in wave reflection may be one potential mechanism behind the risk of acute cardiovascular and cerebrovascular events during acute inflammation.

The continuation of this study in at‐risk populations would provide valuable insights into cardiovascular risk during acute inflammation. In older adults, acute inflammation increases aortic stiffness (Jae et al. 2013), increases peripheral vasodilation (Lane‐Cordova et al. 2016), and reduces total peripheral resistance (Lane et al. 2015), likely altering both wave reflection magnitude and timing. Augmentation index is unchanged in older adults during acute inflammation (Jae et al. 2013; Ranadive et al. 2014); however, wave separation analyses have not been performed and may provide greater insight into mechanisms of cardiovascular and cerebrovascular risk during acute inflammation. It is possible that earlier return of the reflected wave may augment pressure and shift the workload/perfusion balance of the heart, whereas reduced wave reflection magnitude at the carotid could increase transmission of pulsatility into the cerebral circulation. Together, these alterations may be enough to precipitate negative cardiac and cerebrovascular consequences in at‐risk populations with atherosclerosis, hypertension, and other prominent cardiovascular disease risk factors (Lau et al. 2018).

### Limitations

Our measure of aortic PWV was not obtained with the gold standard measurement but instead was estimated from pulse wave and wave separation analyses. Second, we do not have measures of cerebrovascular pulsatile hemodynamics to further expand the implications of our findings although previous literature suggests cerebral blood flow pulsatility may be increased during acute inflammation (de Azevedo et al. 2017). This must be confirmed with future cerebrovascular and carotid blood flow investigations. Furthermore, this study was not powered to detect sex differences in the response to acute inflammation. Exploratory secondary analyses (data not shown) did not reveal any significant sex‐by‐inflammation interactions, suggesting similar effects of inflammation in males and females; however, an adequately powered study would be required to validate these results. The inclusion of females on oral contraceptives could have also altered our findings. However, our exploratory secondary analyses (data not shown) also revealed no effect of oral contraceptives on the female response to inflammation. Finally, our findings are specific to young adults and have limited generalizability to other populations. Further investigation in other populations would provide valuable insights into cardiovascular risk during acute inflammation.

## Conclusion

In conclusion, there were significant reductions in wave reflection at both the aorta and carotid during acute inflammation in young adults, potentially due to downstream/peripheral vasodilation. Acute inflammation‐induced reductions in aortic wave reflections did not disturb the indices of cardiac work/perfusion balance in our sample of young adults; however, the reduced carotid wave reflections suggest changes in cerebrovascular tone that may alter the vulnerability to pulsatile blood flow. These findings may have implications for cardiac and cerebrovascular risk during acute inflammation; however, further research is necessary.

## Conflict of Interests

The authors have no competing interests to disclose.
